# In Vivo and In Vitro Effects of Antituberculosis Treatment on Mycobacterial Interferon-γ T Cell Response

**DOI:** 10.1371/journal.pone.0005187

**Published:** 2009-04-13

**Authors:** Ilaria Sauzullo, Fabio Mengoni, Miriam Lichtner, Anna Paola Massetti, Raffaella Rossi, Marco Iannetta, Raffaella Marocco, Cosmo Del Borgo, Fabrizio Soscia, Vincenzo Vullo, Claudio Maria Mastroianni

**Affiliations:** 1 Department of Infectious and Tropical Diseases, “Sapienza” University, Rome, Italy; 2 Infectious Diseases Unit, “Sapienza” University, Polo Pontino, Latina, Italy; 3 Infectious Diseases, S.M. Goretti Hospital, Latina, Italy; University of Cape Town, South Africa

## Abstract

**Background:**

In recent years, the impact of antituberculous treatment on interferon (IFN)-γ response to *Mycobacterium tuberculosis* antigens has been widely investigated, but the results have been controversial. The objective of the present study was: i) to evaluate longitudinal changes of IFN-γ response to *M. tuberculosis-*specific antigens in TB patients during antituberculous treatment by using the QuantiFERON-TB Gold (QFT-G) assay; ii) to compare the differences in T-cell response after a short or prolonged period of stimulation with mycobacterial antigens; iii) to assess the CD4+ and CD8+ T cells with effector/memory and central/memory phenotype; iv) to investigate the direct in vitro effects of antituberculous drugs on the secretion of IFN-γ.

**Principal Findings:**

38 TB patients was evaluated at baseline and at month 2 and 4 of treatment and at month 6 (treatment completion). 27 (71%) patients had a QFT-G reversion (positive to negative) at the end of therapy, while 11 (29%) TB patients remained QFT-G positive at the end of therapy. Among the 11 patients with persistent positive QFT-G results, six had a complete response to the treatment, while the remaining 5 patients did not have a resolution of the disease. All 27 patients who became QFT-G negative had a complete clinical and microbiological recovery of the TB disease. In these patients the release of IFN-γ is absent even after a prolonged 6-day incubation with both ESAT-6 and CFP-10 antigens and the percentage of effector/memory T-cells phenotype was markedly lower than subjects with persistent positive QFT-G results. The in vitro study showed that antituberculous drugs did not exert any inhibitory effect on IFN-γ production within the range of therapeutically achievable concentrations.

**Conclusions:**

The present study suggests that the decrease in the *M. tuberculosis*-specific T cells responses following successful anti-TB therapy may have a clinical value as a supplemental tool for the monitoring of the efficacy of pharmacologic intervention for active TB. In addition, the antituberculous drugs do not have any direct down-regulatory effect on the specific IFN-γ response.

## Introduction

The immunologic and clinical relevance of measuring T cell response to *Mycobacterium tuberculosis* infection has been highlighted in a wide number of studies. In particular, the development and introduction in clinical practice of the interferon-gamma release assays (IGRAs) has open new perspectives for the detection of tuberculosis (TB) infection [1]–[5]. These assays detect cellular immune response by measuring interferon-gamma (IFN-γ) released by T cells after stimulation by *M. tuberculosis* antigens, such as early secreted antigen target (ESAT)-6 and culture filtrate protein (CFP)-10. These proteins, encoded by genes located within the region of difference 1 (RD1) segment of the *M. tuberculosis* genome, are highly specific for *M. tuberculosis* and are secreted by metabolically active and viable bacilli [6]–[7]. The IFN-γ assay has several advantages over tuberculin skin test (TST), in terms of higher specificity and better correlation with surrogate measures of exposure to *M. tuberculosis* in low-incidence setting, and less cross-reactivity with *M. bovis* Bacille Calmette Guerin (BCG) vaccine strains and environmental mycobacteria [8]–[14].

Despite the growing body of evidence supporting the routinely use of IFN-γ-based tests in clinical practice, there are important issues that need to be thoroughly investigated. In particular, it remains to be clarified the dynamics of specific T-cell responses with the use of IGRAs in patients with active TB before, during, and after antituberculosis treatment. The impact on the IFN-γ responses of antituberculous treatment has been previously examined, but the results have been controversial. In fact, some studies have shown decreasing or negative responses [15]–[19], while others described increased or persistently positive responses during and after treatment [20]–[25]. The variability of the IFN-γ responses is influenced by various factors, including assay characteristics, antigen load in different stage of the disease and the functional diversity of T-cell response. It has been suggested that the short period of incubation (16–24 hours) commonly used in IGRAs detect responses of activated effector T cells that rapidly release IFN-γ when stimulated in vitro with antigen [15], [17], [26]–[28]. By contrast, long-lived central memory T cells may be less likely to release IFN-γ during the short period of exposure to antigen in the IGRA assay, while they could be detected after a longer period of in vitro stimulation [29]–[30]. Since the effector response is driven by the antigen load, it is thought that in the presence of active mycobacterial replication (active untreated TB), the numbers of effector T cells are increased. In the context of a low antigenic load, as in successfully treated patients, the number of effector cells specific to *M. tuberculosis* antigen could fall below the cut-off level [27]. To date, it is unclear if IGRAs may serve as surrogate markers of mycobacterial burden that could be useful for the monitoring of the response to anti-TB treatment.

In this study, we evaluated longitudinal changes of IFN-γ response to *M. tuberculosis-*specific antigens in TB patients during the standard antituberculous treatment by using the QuantiFERON-TB Gold (QFT-G) assay, a commercial IGRA commonly used in clinical practice. The results of QFT-G test were correlated with microbiologic and clinical outcome. Furthermore, to better define the dynamic of immunologic response at the end of treatment, a whole blood IGRA was used to compare IFN-γ secretion after an overnight and a 6-day incubation with mycobacterial recombinant RD1 proteins, respectively. Finally, the impact of the anti-TB treatment on QFT-G result was also evaluated by assessing the direct in vitro effects of antituberculous drugs on the secretion of IFN-γ.

## Materials and Methods

### Study population

The study population included 38 patients with active TB, admitted to the Department of Infectious and Tropical Diseases of the Sapienza University of Rome, Italy. Twenty-six patients had pulmonary TB, while twelve were affected by extrapulmonary disease (three lymphadenitis, one meningitis, four vertebral TB, two disseminated TB and two peritoneal TB). Diagnosis of TB was made on the basis of clinical and radiological findings and was confirmed by identification of *M. tuberculosis* with microbiological methods and/or histological examination of affected tissues. All patients were treated with the four classical antituberculous drugs (rifampicin, isoniazid, pyrazinamide and ethambutol). The control group included 16 healthy unexposed individuals with no history of TB in the past, and no know TB contact. All patients were seronegative for HIV infection. Baseline information for all patients is shown in Table 1. For each enrolled participant venous blood samples were collected in heparin-containing tubes at the time of diagnosis, 2 and 4 months after the beginning of treatment and at completion of specific treatment (6 months). Patients were classified as ‘responders to treatment’ if, after 6 months of specific therapy, the results of microbiological cultures in appropriate specimens were negative for *M. tuberculosis* and there was a clinical and radiological improvement associated with normal inflammatory parameters (C-reactive protein concentration and sedimentation rate). Drug susceptibility testing for rifampicin and isoniazid was performed using the Genotype MTBDR assay (Hain Lifescience, Nehren, Germany). The control group was sampled 3 times at corresponding intervals (every 15 days). The study was approved by the institutional review board (Department of Infectious and Tropical Diseases, Sapienza University of Rome) and informed written consent was obtained before blood donation.

**Table 1:** Baseline characteristics of the subjects included in the study.

Characteristics	Patients with active TB (n = 38)	Healthy control subjects (n = 16)
**Age (range, years)**	15–78	20–65
**Sex**		
Male	18	9
Female	20	7
**Place of birth**		
Italy	20	16
Africa	8	0
Eastern Europe	6	0
Asia	4	0
**BCG vaccination**	10	0
**Active TB**		
Pulmonary disease	26	0
Extrapulmonary disease	12	0

**Abbreviations:**

BCG: Bacillus Calmette and Guerin; TB: tuberculosis.

### Commercial QuantiFERON-TB Gold (Cellestis Limited, Australia)

The QFT-G was performed according to the manufacturer's instructions. Briefly, a blood samples was draw into heparin tubes, and within 2–6 hours of blood draw, four aliquots of 1 ml of heparinized blood were incubated overnight in wells of a plate at 37°C in a humidified atmosphere in the presence of 3 drops of negative control (saline), mitogen positive control (phytohemagglutinin) and two *M. tuberculosis*-specific antigens: ESAT-6 and CFP-10 (overlapping peptides). After 24 hours incubation, plasma aliquots were harvested, and frozen at 70°C, until the assay was performed. The amount of IFN-γ released (IU/ml) was determined using enzyme-linked immunosorbent assay (ELISA).

For each subject, the negative control value was subtracted from the values of antigen-stimulated plasma samples. The cut-off value for a positive response was set at 0.35 IU/ml for ESAT-6 and CFP-10, while for a negative response the concentration of IFN-γ in both antigen wells was less than 0.35 UI/ml and concentration in the positive-control well was 0.5 UI/ml or more. The QFT-G test result was considered indeterminate if the concentration of IFN-γ was less than 0.35 UI/ml in both antigens well and less than 0.5 UI/ml in the positive-control well, or if the concentration of IFN-γ in the negative well is higher than 8.0 UI/ml. Analysis of data was done by the QuantiFERON-TB Gold Analysis Software.

### Detection of IFN-γ release after overnight and a 6-day incubation with mycobacterial antigens

The production of IFN-γ release after overnight and a 6-day incubation with mycobacterial antigens was performed using a whole blood IGRA. Heparinized blood was diluted 1 in 5 with serum-free medium (RPMI supplemented with penicillin and streptomycin plus 2 mM L-glutamine) and 250 µL was seeded in a well plate and were stimulated with following stimuli: i) intact recombinant (r)ESAT-6 protein (Lionex, Germany: purity >95%; endotoxin content: 655 EU/mg;) at 1 µg/mL and (r)CFP-10 protein (Lionex, Germany: purity >95%; endotoxin content: 9,8 EU/mg;) at 0.5 µg/ml; ii) purified protein derivative (PPD; batch RT47; Statens Serum Institute) at 5 µg/mL; iii) phytohemagglutinin (PHA) (Sigma, St Louis, MO, USA) at 5 µg/mL as positive control; iv) medium alone as negative control. The samples were incubated on the day of blood collection at 37°C with 5% CO_2._ Supernatants were harvested after overnight and 6-day incubation and then were immediately tested for IFN-γ by using a commercial ELISA, according to the manufacturer's instructions (QuantiFERON-CMI, Cellestis Ltd, Carnegie, Victoria, Australia). IFN-γ concentrations were expressed as IU/ml, and the negative control value was subtracted from the values of antigen-stimulated plasma samples.

### Phenotypic analysis by flow cytometry

Phenotypic analysis of CD4+ and CD8+ T cells was performed by flow-cytometric analysis, analyzing expression of the memory markers such as CD45RO and CCR7.

Heparinized whole blood was diluted, stimulated and incubated as described above. After 6 days of incubation, supernatants were recovered and the cells were harvested by washing the wells with 300 µl of PBS. The following monoclonal antibodies (mAbs) were added: anti-CD4-peridinim chlorophyll (PerCP); anti-CD8-peridinim chlorophyll (PerCP); anti-CD45RO-allophycocyanine (APC); anti-CD3-fluorescein isothiocyanate (FITC), and anti-CCR7-phycoerythrin (PE). All mAbs were purchased from Becton Dickinson (BD Biosciences Pharmigen, Italy). The cells were incubated for 20 minutes in the dark at room temperature (RT); 1 ml of FACS Lysing Solution (BD) was added, vortexed and incubated for 15 minutes at RT. Samples were analyzed within 1 h of the staining using a FACSCalibur flow cytometer and CellQuest software version *1.0* (Becton Dickinson, Mountain View, CA, USA). All data were collected using identical instrument settings.

We defined an R1 gate for lymphocytes in a dot plot of Forward Scatter Chanel (FSC) *versus* Side Scatter Chanel (SSC). To identify CD4+ and CD8+ T cells, events from R1 were analyzed in a plot of CD3-FITC *vs* CD4-PerCP or CD8-PerCP respectively (R2). Finally, gated CD4+ and CD8+ T cells were analyzed for CCR7-PE *vs* CD45RO-APC.

Data are reported as percentages of CD4+ and CD8+ T cells with both an effector/memory (EM) phenotype, defined as CD45RO+/CCR7−, and central/memory (CM) phenotype, characterized by the CD45RO+/CCR7+.

### In vitro effect of antituberculous drugs on the IFN-γ release

The in vitro effect of the combination of rifampicin (RIF)/isoniazid (INH)/pyrazinamide (PZA)/ethambutol (ETB) (Becton Dickinson) on the IFN-γ release was performed as follows. Aliquots of 0,5 ml of heparinized blood were incubated overnight at 37°C, with 5% CO_2_, in the presence of PHA (5 µg/mL) or a solution of the 4 antituberculous drugs at three different concentrations. On the basis of therapeutically achievable concentrations, the first concentration (C1) of combined drugs was: INH 5 µg/ml, RIF 7 µg/ml, ETB 5 µg/ml, PZA 40 µg/ml. The other concentrations were two (C2) or three (C3) times greater. All conditions were set up in triplicate wells. Positive control wells contained only PHA at 5 µg/ml. The IFN–γ release (expressed as IU/ml) in the presence or absence of the drug, was assessed by ELISA, using a commercial kit, according to the manufacturer's recommendations (QuantiFERON-CMI, Cellestis Ltd, Carnegie, Victoria, Australia). Cellular vitality was assessed with trypan blue.

### Statistical analysis

SPSS version 13.0 for windows (SPSS Inc., *Apache Software Foundation*, Chicago, Illinois) was used. The differences of values between the different groups were analysed using the non-parametric Mann-Whitney *U*-test. Data in the longitudinal analysis during the treatment course of individual patients were evaluated with the non parametric Wilcoxon signed-rank test (two tailed). Student's *t* test was used for statistical analysis of data from in vitro experiments with antituberculous drugs. *P*–value <0,05 was regarded as significant.

## Results

### Changes in the T-cell IFN-γ response to *M. tuberculosis* antigens during anti-TB therapy by QFT-G assay

Thirty eight patients with confirmed TB were followed up longitudinally and tested with QFT-G at the time of diagnosis, 2 and 4 months after the beginning of treatment and at completion of specific treatment (6 months).

At baseline all 38 patients had positive QFT-G responses. After the beginning of anti-TB treatment, QFT-G assay was positive in 31/38 (81.5%) patients at month 2 and in 22/38 (57.8%) patients at month 4. At the end of therapy a reversion to negative response was found in 27/38 (71%) of patients, while 11/38 (29%) patients remained QFT-G positive.

**Figure 1:**
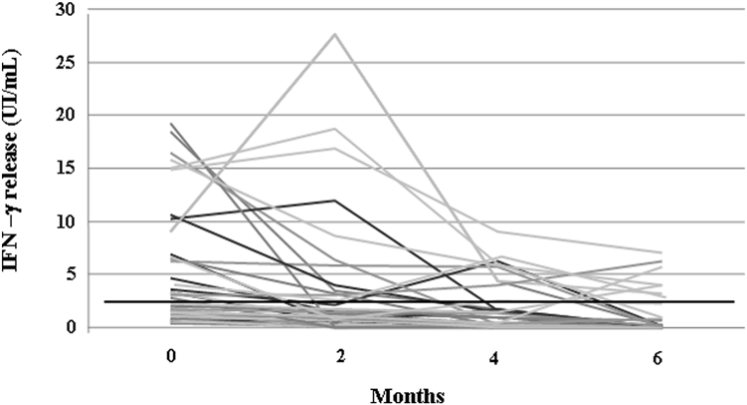
Longitudinal changes of specific IFN-γ response in 38 TB patients following anti-tuberculous treatment. IFN-γ response to sum of ESAT-6 and CFP-10 (overlapping peptides) was measured by QFT-G before, during and at the end of treatment. A significant decrease of IFN-γ response to mycobacterial antigens was found during the therapy (p<0.001, Wilcoxon's signed-rank test). [p: for the comparison of the results at baseline vs. completion of therapy]. Horizontal black line indicates the QFT-G assay cut-off value for a positive result (0.35 UI/mL).

The individual change of IFN-γ response to sum of ESAT-6 and CFP-10 peptides during the treatment for each participant is shown in Figure 1. Taken together, the data showed a significant decrease in average IFN-γ response in all 38 TB patients during the follow-up (p<0.001). The median of IFN-γ concentrations in the 27 patients who became QFT-G negative throughout the 6 months of treatment and in the 11 patients who remained persistently QFT-G positive on treatment completion was reported in Table 2. The results showed that there was a significant decrease in IFN-γ concentrations in TB patients who became QFT-G negative at the end of treatment (p<0.001). By contrast, after an initial decline, the concentrations of IFN-γ did not show significant changes in patients who remained QFT-G positive on treatment completion (p = 0,085). No significant differences were seen in the baseline IFN-γ concentrations between the two groups of patients (p = 0,215).

**Table 2:** IFN-γ concentrations in TB patients according to the results of QFT-G assay at the end of treatment.

Patients	Median IFN-γ release in UI/mL (range)				*P* value
	0 months	2 months	4 months	6 months	
**Group A** (n = 27)					
ESAT-6/CFP-10	2,79 (0,41–19,29)	0,82 (0–27,67)	0,21 (0–6,22)	0,02 (0–0,3)	<0,001
**Group B** (n = 11)					
ESAT-6/CFP-10	3,63 (1,44–15,77)	2,1 (0,82–27,67)	4 (0,4–9,05)	3,25 (0,68–7)	0,085

**Group A:** TB patients who had a QFT-G reversion (positive to negative) at the end of therapy.

**Group B:** TB patients who remained QFT-G positive at the end of therapy.

All patients who showed a significant decline in IFN-γ concentrations and became QFT-G negative after treatment had a complete clinical and microbiological recovery of the TB disease. Among the 11 patients with persistent positive QFT-G results, 6 patients were considered as ‘responders to treatment’ on the basis of clinical, radiological, microbiological and laboratory findings; the remaining 5 patients, who had persistent culture positive samples after 2 months, did not have a complete resolution of the clinical disease and early relapsed after the 6-month treatment completion (Table 3).

**Table 3:** Characteristics of 5 TB subjects with persistent positive IGRA results and less good outcome.

Patients	Time of relapse after treatment completion	Drug resistance	Ultimate clinical outcome
**1**	10 weeks	INH+RIF	Cured after 12 months of therapy
**2**	8 weeks	INH	Lost at follow-up
**3**	8 weeks	INH+RIF	Cured after 12 months of therapy
**4**	4 weeks	ND	Disseminated disease
**5**	6 weeks	INH+RIF	Cured after 9 months of therapy

**Abbreviations:**

ND: not done; INH : Isoniazid; RIF: rifampicin.

To determine whether the changes in response to specific antigens were indicative of *M. tuberculosis*–specific immunity and not the result of generalized fluctuations in T-cell response, we also carried out a longitudinal analysis in 16 healthy controls. There was no significant variations in IFN-γ concentrations during longitudinal QFT-G testing at 3 different intervals of time (every 15 days) [median IFN-γ (range): 0.01 UI/mL (0–0.11); 0.01 UI/mL (0.1–0.13); 0.015 UI/mL (0–0.1); p = 0,664]. Thus, this group of healthy unexposed individuals with no history of TB and no know TB contact maintained stable the IFN-γ concentration below the detection threshold of the ex vivo QFT-G.

### IFN-γ production in response to *M. tuberculosis* antigens using different incubation times

The differences in T-cell response after a short or prolonged period of stimulation with mycobacterial antigens was investigated in a subset of 8 TB patients who had a QFT-G reversion (positive to negative) after completion of treatment and in 10 TB subjects who remained QFT-G positive at the end of therapy. IFN-γ concentrations in response to sum of rRD1 proteins (rESAT-6 and rCFP-10), PPD and PHA were measured after overnight and 6-day incubation by a whole blood IGRA (Table 4).

**Table 4:** IFN-γ responses to mycobacterial antigens, PPD and PHA assessed by short or prolonged incubation-based assays.

Patients	Median IFN-γ release in UI/mL (range)		*P* value
	Incubation time 18 h	Incubation time 6 days	
**Group A** (n = 8)			
rESAT-6/rCFP-10	0 (0–0,83)	0,015 (0–0,19)	0,625
PPD	0,865 (0–8,3)	7,2 (0,04–8,29)	0,033
PHA	2,65 (0,21–8,25)	8,55 (2.19–16,93)	0,6
**Group B** (n = 10)			
rESAT-6/rCFP-10	2,5 (0,02–7)	6,69 (2,17–17,95)	0,004
PPD	5,16 (0,43–18,21)	8,44 (5,19–18,55)	0,005
PHA	7,39 (1,16–19,76)	9,19 (1,08–20,1)	0,5
**Healthy controls** (n = 5)			
rESAT-6/rCFP-10	0 (0–0,36)	0 (0–0,05)	0,211
PPD	0,15 (0,1–0,44)	0,29 (0,15–0,51)	0,078
PHA	6,05 (1,05–8,05)	8,27 (0,3–8,89)	0,5

**Abbreviations:**

PPD: purified protein derivative; PHA: phytohemagglutinin; rESAT-6: recombinant ESAT-6; rCFP-10: recombinant CFP-10.

**Group A:** TB patients who had a QFT-G reversion (positive to negative) at the end of therapy.

**Group B:** TB patients who remained QFT-G positive at the end of therapy.

In the 8 patients who became QFT-G negative at the end the treatment, no significant differences in immunologic response were found between short and longer incubation with rRD1 antigens (p = 0,625). Indeed, also the prolonged 6-day in vitro stimulation with rRD1 antigens failed to evoke IFN-γ production, while there was an increased response to PPD (p = 0.03 versus overnight incubation).

By contrast, in the 10 TB patients who remained QFT-G positive after the treatment, a response to all the antigens used was found both after a short and a longer period of stimulation in vitro. The results confirmed that the immune response to each antigens after 6-day incubation was statistically higher compared to overnight stimulation (p = 0,004 for rRD1 and p = 0,005 for PPD) (Table 4). IFN-γ responses to recombinant RD1 proteins after 18 h of stimulation in whole blood IGRA did not differ significantly from those generated with a pool of overlapping peptides used in QFT-G assay (p = 0,733 for ESAT-6 and p = 0,809 for CFP-10 (data not shown).

No significant increase in IFN-γ concentrations was observed in 5 healthy controls in response to any antigens after stimulation of 6 days, although a positive response to the mitogen PHA was detectable (p = 0.211 for rRD1 and p = 0.078 for PPD) (Table 4).

**Figure 2:**
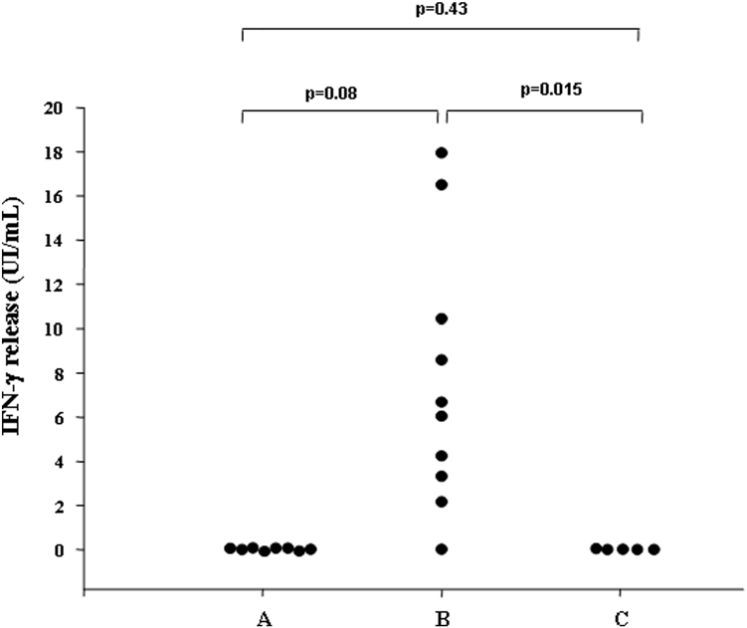
Evaluation of individual IFN-γ production by prolonged-based incubation assay. Response to sum of intact recombinant ESAT-6 and CFP-10 proteins after 6 days of stimulation was evaluated in 8 TB patients with QFT-G reversion (positive to negative) during treatment (A), 10 TB patients with persistent QFT-G positive results at the end of therapy (B), and 5 healthy donors (C). The largest increase in IFN-γ production was seen only in TB patients with persistent positive QFT-G results. Statistical significance was analyzed by the Mann–Whitney U-test.

In summary, our results showed that the increase in IFN-γ production in response to rRD1 proteins after 6 days of stimulation was entirely restricted to the TB patients with persistent positive QFT-G results (Figure 2).

### Phenotypic analysis of CD4+ and CD8+ T cells

The cytofluorimetric analysis of the phenotypic profile of CD4+ and CD8+ T cells in the 10 patients with persistent QFT-G positive results and in the 8 individuals who showed a QFT-G reversion (positive to negative) at the end of treatment is reported in Table 5. In the patients with positive QFT-G results at the end of therapy, the percentage of CD4+ T cells with both effector memory (EM) and central memory (CM) phenotype after 6 days of rRD1 antigen-specific in vitro stimulation was significantly higher than that seen in subjects who showed a QFT-G reversion (p = 0.001 and p = 0.003, respectively).

**Table 5:** Percentage of CD4+ and CD8+ T cells with effector/memory and central/memory phenotype in 18 TB patients at treatment completion.

	Patients		*P* value
	Group A (n = 8)	Group B (n = 10)	
**CD4+ T cells**			
T_EM_% (range)	21,27 (2,61–27,1)	41,7 (12,36–52,65)	0,001
T_CM_% (range)	2,36 (1,69–15,9)	15,82 (11,25–28,39)	0,003
**CD8+ T cells**			
T_EM_% (range)	11,2 (5,6–21,4)	18,55 (12,5–35,6)	0,036
T_CM_% (range)	9,9 (3,1–14)	14 (6,7–22)	0,087

**Abbreviations:**

**T_EM_:** T cells with an effector/memory phenotype, defined as CD45RO+/CCR7−.

**T_CM_:** T cells with central/memory phenotype, defined as CD45RO+/CCR7+.

**Group A:** TB patients who had a QFT-G reversion (positive to negative) at the end of therapy.

**Group B:** TB patients who remained QFT-G positive at the end of therapy.

**Figure 3:**
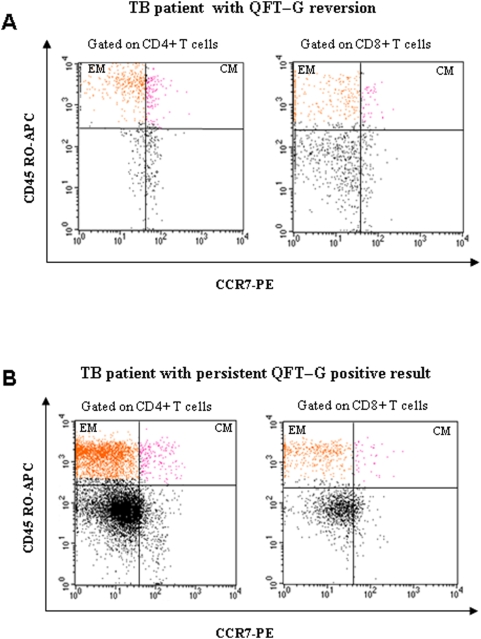
Cytofluorimetric analysis of phenotypic profile of T cells from two representative TB patients at the end of treatment. A representative sample of one TB patient with QFT-G reversion (positive to negative) at the end of therapy (panel A) and one TB patient with persistent QFT-G positive result (panel B) is shown. The percentage of CD4+ and CD8+ T cells that expressed CD45RO and lymphotropic chemokine receptor CCR7 was assessed in diluted whole blood after 6 days of in vitro stimulation with intact recombinant ESAT-6 and CFP-10 proteins. Effector memory (EM) cells (CD45RO+/CCR7−) are shown in the upper left quadrants of both panels; central/memory (CM) cells (CD45RO+/CCR7+) are showed in the upper right quadrants of both panels.

Similarly, the percentage of CD8+T-cells with EM phenotype was significantly higher in patients with positive QFT-G results than in those with negative QFT-G results (p = 0.036 for EM). No significant difference was observed in the percentage of CD8+ T-cells with CM phenotype among two groups of patients (p = 0.087) (Table 5). The cytofluorimetric analysis from a representative TB patient with QFT-G reversion (positive to negative) and a patient with persistent QFT-G positive after treatment is shown in Figure 3.

### In vitro effect of antituberculous drugs on IFN–γ release

**Figure 4:**
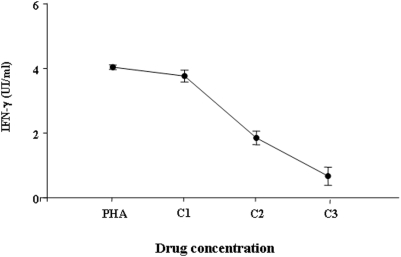
Effect of antituberculous drugs on IFN-γ release in vitro. The IFN-γ production was evaluated after overnight incubation with the combination of four drugs (rifampicin, isoniazid, pyrazinamide, ethambutol) at three different concentrations of solution. C1: INH 5 µg/ml, RIF 7 µg/ml, ETB 5 µg/ml, PZA 40 µg/ml; C2: INH 10 µg/ml, RIF 14 µg/ml, ETB 10 µg/ml, PZA 80 µg/ml; C3: INH 15 µg/ml, RIF 21 µg/ml, ETB 15 µg/ml, PZA 120 µg/ml. Controls wells contained only PHA at 5 µg/ml. The concentrations of IFN-γ produced in the presence of drug concentrations compatible with those achieved in the serum of treated patients (C1) were not significantly different from controls containing only PHA (p = 0,071). In contrast, a significant inhibitory effect was found at more elevated drug concentrations (C2 and C3) (p<0.001 for both). Student's t test was used for statistical analysis.

To determine whether antituberculous drugs had an inhibitory effect on the IFN-γ release by T cells, the heparinized blood samples were incubated in vitro with a combination of INH, RIF, ETB, STR. The concentrations of IFN-γ (mean±SE, UI/ml) measured at the three different used concentrations were as follows: C1, 3.767±0.18; C2, 1.85±0.21; C3, 0.667±0.286 (Figure 4). IFN-γ release was inhibited in a dose-dependent manner. It is important to point out that a significant inhibitory effect was seen only at more elevated drug concentrations if compared to controls containing only PHA (4.04±0.07) (p<0.001, for both C2 and C3). On the other hand, when antituberculous drugs were used at concentrations which are compatible with those achieved in the serum of treated patients (C1), the concentrations of IFN-γ were not significantly different from controls (p = 0.071). No evidence of toxic effect of the combined drugs on the cellular survival was found (data not shown).

## Discussion

In recent years, a number of studies have been conducted to investigate the impact of antituberculous treatment on specific IFN-γ response as measured by QFT-G assay. The analysis of the literature data has shown controversial results in the case of both latent and active TB treatment. Indeed, some authors have demonstrated that IFN-γ response to specific mycobacterial antigens decreased or became negative [15]–[19], while others have reported persistently positive or even stronger responses during and after anti-TB treatment [20]–[25]. It has been suggested that the progressive decline of IFN-γ response measured by QFT-G test reflects the reduction of mycobacterial burden following a successful antituberculous treatment. However, it is unclear if the persistence of IFN-γ responses detected in IGRA assay will be predictive of clinical and microbiological treatment failure or relapse.

The present longitudinal study was designed to evaluate the impact of antituberculous treatment on IFN-γ response in patients with active TB who were followed-up for 6 months before and at completion of therapy. In agreement with our previous results, the QFT-G assay showed an excellent degree of sensitivity and specificity for diagnosis of active TB in our selected patients [31]. In addition, the concentrations of IFN-γ were below the detection threshold of the ex vivo QFT-G in all healthy unexposed individuals with no history of TB and no know TB contact.

The longitudinal analysis by QFT-G assay showed that in the majority of our TB patients there was a correlation between clinical treatment outcome and changes of IFN-γ response to *M. tuberculosis*-specific antigens. Indeed, for all patients who had a complete resolution of clinical disease and whose microbiological culture results were negative for *M. tuberculosis* at 6-month treatment completion, a progressive decline of IFN-γ release was seen and the baseline positive QFT-G result had turned negative. This data are in agreement with previous studies which have reported a progressive decrease in the frequency of *M. tuberculosis* antigen-specific IFN-γ-secreting T cells after successful treatment for active TB [15]–[19], [32]. In a recent study in Gambia, a significant proportion of patients who successfully completed a course of standard TB treatment changed from a positive to a negative IGRA after 12 months after their initial diagnosis [18]. Similarly, in Cape Town, 81% HIV-negative patients who had successfully completed TB treatment were IGRA negative [19]. It has been suggested that negative IFN-γ release assay results after treatment of active TB indicates successful antibiotic-induced killing of all bacilli.

We have also shown that, in the patients with successful treatment for active TB, the release of IFN-γ is absent even after a prolonged 6-day incubation with both ESAT-6 and CFP-10 antigens. It has been suggested that IGRAs based on a prolonged period of incubation with mycobacterial antigens could detect response of central memory T-cells [24] and are more sensitive to identify past latent TB infection; on the other hand, short-incubation IGRAs mainly detect circulating effector memory T cells whose numbers correlate with recent or ongoing active *M. tuberculosis* infection [33]. Our results showed that the percentage of both CD4+ and CD8+ T-cells EM phenotype was markedly lower in the patients whose baseline positive QFT-G result had turned negative at treatment completion. The overall low percentage of T cells with EM phenotype and the progressive disappearance of IFN-γ responses after both short and prolonged stimulation with rRD1 proteins may be consistent with cessation of antigen stimulation in vivo and the clearance of viable bacilli. In the present study, we did not measure the separate IFN-γ responses to ESAT-6 and CFP-10 antigens, although recent findings suggest that quantitative response to CFP-10 only antigen may be a more useful monitoring marker of clinical efficacy for active TB disease treatment [34], [35].

To exclude that the progressive decline of IFN-γ response could be due to a direct inhibitory effect of antituberculous drugs, cells were incubated in vitro with serial concentrations of INH, RIF, ETB and PZA. We demonstrated for the first time that the four antituberculous drugs did not exert any down-regulatory effect on IFN-γ production within the range of therapeutically achievable concentrations. Preliminary works suggest that reversions of IGRA from positive to negative results can occur with serial testing [36]; moreover, T cell responses, especially weakly positive responses, tend to fluctuate over time, even in the absence of specific treatment [37]–[40]. Although the present study was not designed to evaluate the reproducibility of the QFT-G assay, we demonstrated no significant variations in IFN-γ concentrations over time in a group of healthy unexposed individuals with any history of TB and no know TB contact. Overall, these findings suggest that the decrease of IFN-γ response to mycobacterial antigens in patients with successful treatment response is not due to a direct inhibitory of antituberculous drugs or to the variability of the IGRA, but it likely reflects the reduction in bacterial burden.

Nearly one-third of our TB patients were still positive by QFT-G assay despite active TB disease treatment. Increased IFN-γ response was also maintained by using a whole blood IGRA based on a prolonged 6-day in vitro stimulation with mycobacterial antigens. In these patients with persistent positive response on QFT-G assay most CD4+ T-cells had an EM phenotype. In one recent study, Katyar et al. suggested that a higher IFN-γ response at 2 months could be an independent indicator of the likelihood of remaining sputum culture-positive at the end of the intensive phase of anti-tuberculosis treatment [32]. In another study, Carrara et al. suggested that a delayed drop in TB IFN-γ release could be an indicator of adverse outcome and poor response to treatment [17]. In the present study, 5 of the 11 patients with persistent positive IGRA response did not respond to the 6-month treatment course, whereas the remaining six patients had clinical and microbiological response to treatment. The reasons for the persistence of increased IFN-γ response in spite of a successful treatment for active TB are not yet established. Since our patients come from a country with low TB incidence (Italy), it is reasonable to rule out a possible continued exposure to *M. tuberculosis*. On the other hand, we cannot exclude potential exposure to environmental mycobacteria, as well as persistent T-cell response in the presence of low antigen stimulation or genetic polymorphisms in the host.

In conclusions, the present study suggests that the decrease in the *M. tuberculosis*-specific T cells responses following successful anti-TB therapy may have a clinical value as a supplemental tool for the monitoring of the efficacy of pharmacologic intervention for active TB. In addition, the antituberculous drugs do not have any direct effect on host immune response. Investigations on T-cell kinetics and functions are needed to better investigate the significance of the persistent IGRA positive results which are not predictive of a negative clinical outcome and poor response to treatment.
